# AtlasGrabber: a software facilitating the high throughput analysis of the human protein atlas online database

**DOI:** 10.1186/s12859-022-05097-9

**Published:** 2022-12-17

**Authors:** Benedek Bozoky, Laszlo Szekely, Ingemar Ernberg, Andrii Savchenko

**Affiliations:** 1 Department of Microbiology, Tumor and Cell Biology (MTC), Biomedicum C8, Solnavägen 9, 171 65 Solna, Sweden; 2 Department of Laboratory Medicine, Karolinska Institutet, Alfred Nobels Allé 8, 171 77 Huddinge, Sweden

**Keywords:** Human protein atlas, AtlasGrabber, Immunohistochemistry, Protein expression, Tissue microarray, Biomarkers, Prostate cancer, Basal cells

## Abstract

**Background:**

The human protein atlas (HPA) is an online database containing large sets of protein expression data in normal and cancerous tissues in image form from immunohistochemically (IHC) stained tissue microarrays. In these, the tissue architecture is preserved and thus provides information on the spatial distribution and localization of protein expression at the cellular and extracellular levels. The database is freely available online through the HPA website but currently without support for large-scale screening and analysis of the images in the database. Features like spatial information are typically lacking in gene expression datasets from homogenized tissues or single-cell analysis. To enable high throughput analysis of the HPA database, we developed the AtlasGrabber software. It is available freely under an open-source license. Based on a predefined gene list, the software fetches the images from the database and displays them for the user. Several filters for specific antibodies or images enable the user to customize her/his image analysis. Up to four images can be displayed simultaneously, which allows for the comparison of protein expression between different tissues and between normal and cancerous tissues. An additional feature is the XML parser that allows the extraction of a list of available antibodies, images, and genes for specific tissues or cancer types from the HPA’s database file.

**Results:**

Compared to existing software designed for a similar purpose, ours provide more functionality and is easier to use. To demonstrate the software’s usability, we identified six new markers of basal cells of the prostate. A comparison to prostate cancer showed that five of them are absent in prostate cancer.

**Conclusions:**

The HPA is a uniquely valuable database. By facilitating its usefulness with the AtlasGrabber, we enable researchers to exploit its full capacity. The loss of basal cell markers is diagnostic for prostate cancer and can help refine the histopathological diagnosis of prostate cancer. As proof of concept, with the AtlasGrabber we identified five new potential biomarkers specific for prostate basal cells which are lost in prostate cancer and thus can be used for prostate cancer diagnostics.

## Background

Numerous gene expression datasets are available from homogenized tissues, cell lines, and single cells. However, these sources do not provide conclusive information on cell type, subcellular protein localization, extracellular matrix (ECM) proteins, or the distribution of expression in tissues. Recently, efforts to characterize distinguishing features in whole human cellular populations have been undertaken. Still, these are limited by being based on already established cellular markers [1, 2].

**Fig. 1:**
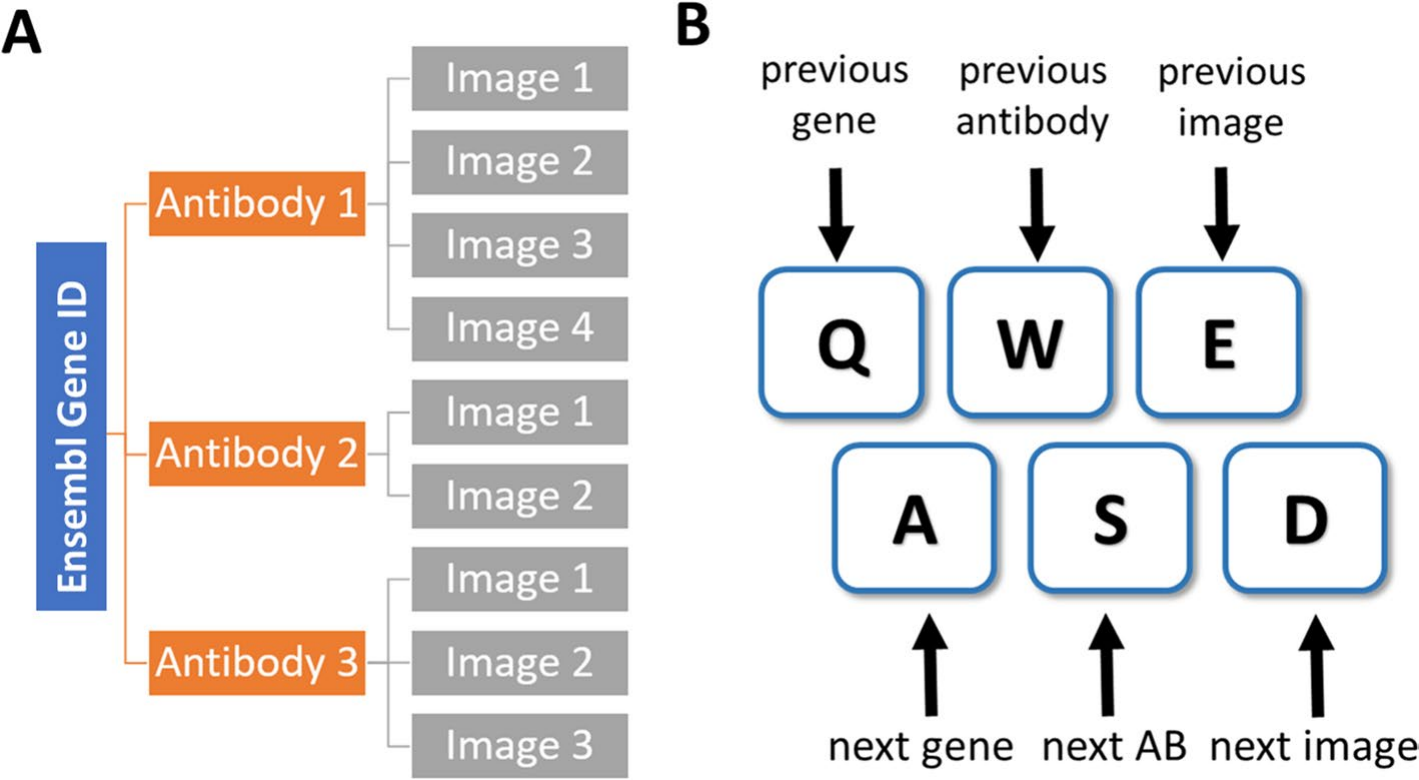
**A** Schematic presentation of the organization of images in the HPA. Several antibodies target each gene, and for each antibody, one can retrieve several images with stainings. The AtlasGrabber helps to organize the analysis of these images in a desktop application. **B** Shows the key bindings that can be used to scroll through the images

The Human Protein Atlas (HPA) [3–6] is an online, open-access database that contains over 10 million high-resolution images of tissue microarrays with immunohistochemical (IHC) stainings. It includes both normal and cancerous tissues. In this way, the HPA shows protein expression in the form of images of immunohistochemically stained tissue samples. The current version (version 21.0) covers 44 different normal tissues, the 20 most common cancer forms, and includes more than 87% of the human proteins. There are usually multiple antibodies (1–3) targeting each protein in the database (Fig. 1A). Each gene is typically targeted with an in-house generated (from the HPA project) and a commercial antibody. This data is freely accessible via the HPA website (https://www.proteinatlas.org).

The database’s main advantage is that the images contain information on the expression, spatial distribution, and localization of each protein in the different tissues. Thus, the HPA can be used for better spatial localization of protein expression than any other resource can provide. It provides the protein expression patterns in single-cell types and subtypes, localization inside cells, tissues, and ECM and thereby provides a database of such characteristics of biomarker expression. This is particularly important information in cancer tissues, as cellular plasticity and changes in the tumor microenvironment are emerging as key factors in cancer pathogenesis, progression, and cell invasiveness. These tissue features show meaningful connections to most canonical cancer hallmarks [7–10]. The HPA can be regarded as a hypothesis-generating tool, as a supplement to other high throughput (HTP) expression data, and provides a basis for experimental approaches [11, 12].

The atlas has a freely accessible user-friendly website for exploration. However, it does not directly support direct and fast large-scale HTP analysis, and it doesn’t allow for the easy comparison of the gene expression between normal and cancerous tissues, and between different tissue types. Realizing the potential of exploiting the atlas for extensive HTP analysis, we developed an application designated as AtlasGrabber to enable this. Researchers can use our tool to analyze a set of genes of interest for protein expression in different normal and cancerous tissues, comparing and sorting them into separate sets. The software can also provide information on the number of available genes, specific antibodies, and image links for each normal or tumor tissue in the HPA XML database file [13]. Here we describe the software and demonstrate its usefulness by identifying novel immunohistochemical biomarkers for prostate basal cells, comparing their expression in normal and cancerous tissues. With the expansion of digital pathology, additional IHC based tissue repositories will be established, for which a similar approach can be adopted.

## Implementation

The code was written in the C# programming language as a Windows Desktop application. The code is open source under the Gnu Public License v3 [14] and freely available on GitHub [15]. Users can download the source code to compile it or download and run an available executable.

The AtlasGrabber’s intended use is to facilitate the analysis of the protein expression in the HPA from a set of genes. It does so by displaying the images from the HPA in an organized, systematic way, based on a gene list, and allows the saving of genes of interest into new subsets. It is possible to simultaneously analyze a set of predefined genes in up to four different tissue types in normal or cancerous tissues. The gene set analyzed may contain thousands of genes and allows the comparison between stainings with the same antibody in different tissues. An additional feature is the XML parser, which can extract all the gene names, antibodies, and images for a particular tissue from the XML file provided on the HPA website.

An initial text file (.txt) that contains a list of Ensembl IDs for the genes the investigator intends to analyze is needed to start using the AtlasGrabber. Such lists can also be generated from the downloadable files from the HPA website (http://www.proteinatlas.org/about/download) or by searching keywords in the HPA search field and exporting the file. Detailed step-by-step video instructions can be found in the Readme file on GitHub [15].

The software executable can be downloaded directly [15] or compiled from the source code. No additional setup or installations are required. The software has been tested to run on Windows 8, 10, and 11. We recommend using a high-definition, large-screen monitor (above 20 inches) for the best experience as the software will maximize the usage of the screen area by recalculating the area occupied by each window depending on the screen area.

**Fig. 2:**
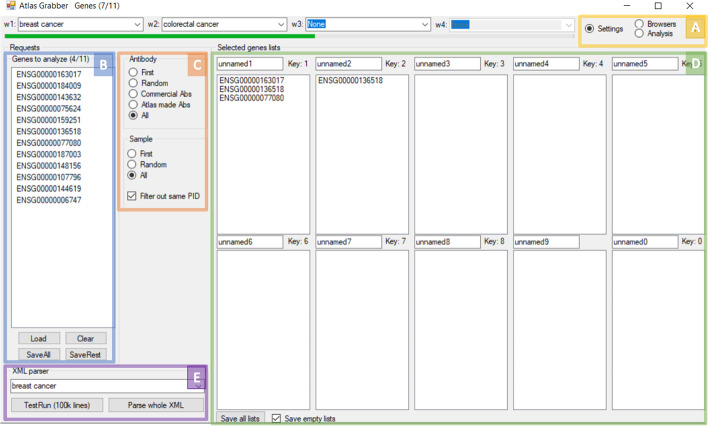
AtlasGrabber user interface with the Settings view open. **A** The three window options: settings, browsers, and analysis. **B** Allows for the loading and saving of a gene list in the form of Ensembl IDs from a text file. **C** Options for selecting the images with the possibility to filter images based on antibodies, all images, just a random image, or the first image, and if to filter images from the same patient. **D** The gene set view allows the naming of the lists and saving lists. **E** The XML parser can extract all Ensembl IDs for a particular tissue, normal or cancerous

The program uses three different windows: settings, browsers, and analysis windows (Fig. 2A). Initially, the program opens to the “Settings” window (Fig. 2). Here one can load the gene list from the text file (Fig. 2B). Additional options include the possibility to specify the analysis to all antibodies or to separate commercial and in-house ones, to look at all the image samples, just one, or a random one, and to filter away additional images from the same patient sample for one antibody (typically there are two images per patient sample) (Fig. 2C). In this window, it is also possible to name different lists for the storage of selected genes (Fig. 2D). Each list is assigned a key: from 0 to 9. While in the Analysis window, looking through the atlas, the current gene ID is copied to any of the ten lists with the assigned key. If saved, the list will appear in the same folder where the program is located. If the file already exists from a previous analysis, the new gene names will be added to the old ones in case one chooses to do the analysis in multiple runs.

**Fig. 3:**
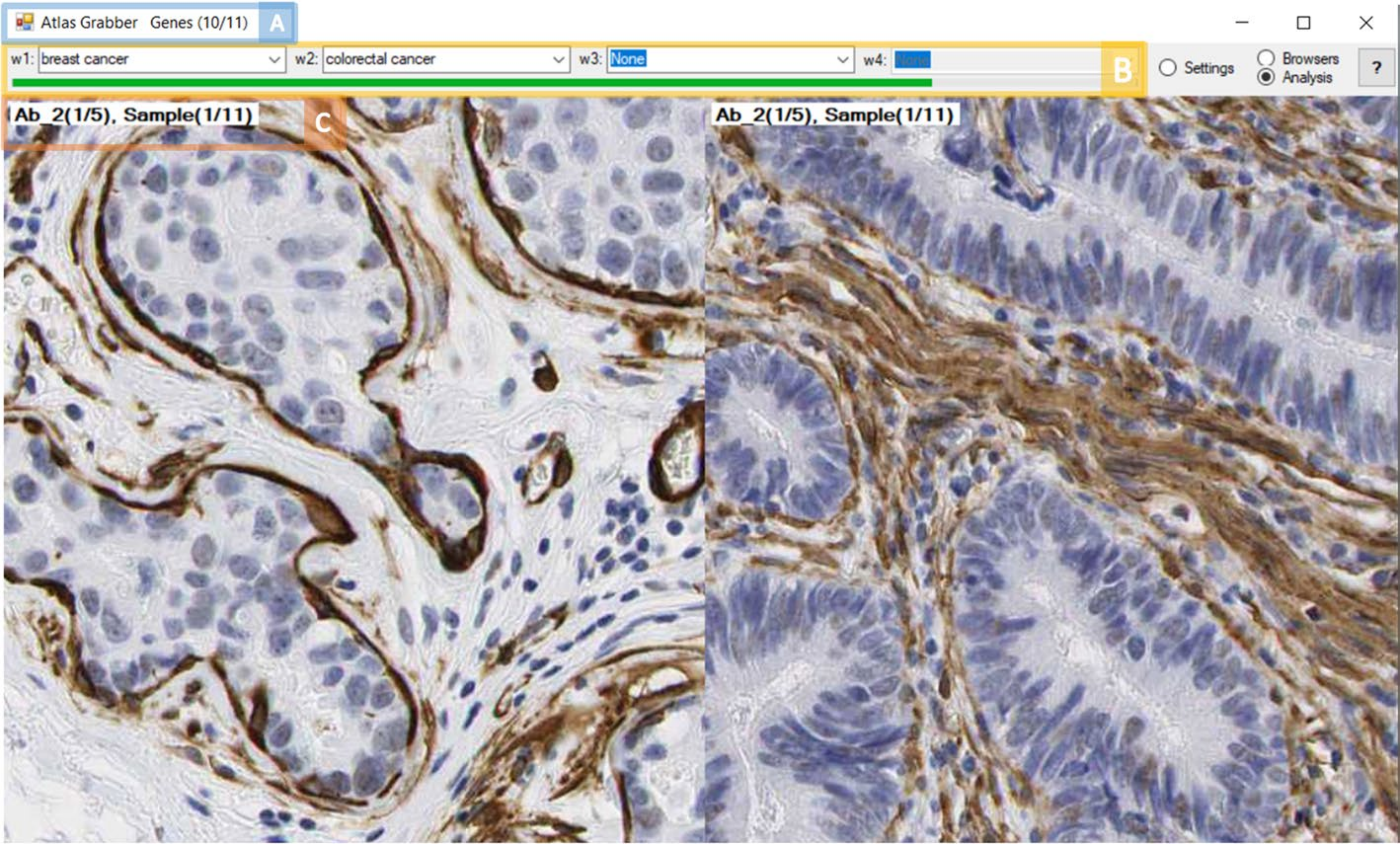
Display of the Analysis window for performing the image analysis, in this example, for smooth muscle alpha-actin in breast cancer (left) and colorectal cancer (right). **A** The number of genes analyzed is displayed next to the title. **B** Allows for the viewing of multiple image windows. Each window can be a different tissue: normal or cancerous. The green progress bar indicates the progress of the analysis. **C** Information about the antibody for each image: Number of antibodies (out of total) and image sample number (out of total). Image credit: human protein atlas

The tissues to be analyzed are selected at the top of the screen. One can choose any normal or cancer tissue from the dropdown menu in any of the four menu windows. When a new window is assigned to a tissue, this new window will be added in the Analysis view (Fig. 3).

To start loading and viewing images, the user selects the “Analysis” window (Fig. 3). Images will be displayed for each antibody in the gene list. The mouse enables the spanning of the image. We recommend using the assigned keyboard keys to move through the images, antibodies, and proteins (Fig. 1B). The scrolling wheel can also be used to move through the images. Pressing any of the keys 0–9 will assign the gene ID to be saved to that list. Returning to the “Settings” window, one can see which gene (ID) is currently being analyzed in the left panel and which gene IDs have been assigned to the different lists.

The “Browsers” window will display the HPA website of the particular antibody in a web browser. This window can be used to read a quick summary about the gene or the antibody. For example, if during the analysis the user identifies an interesting antibody candidate, they can quickly access the HPA information on the antibody, (e.g. antibody provider, antibody validation) and protein summary e.g. names, alternative names, description, intracellular location etc.). This window can also be used as a debug mode. The progress bar at the top of the screen will show the progress of the analysis (Fig. 3B), and the exact gene number from the list is displayed in the application’s title (Fig. 3A). The Help button links to the Readme file on the GitHub page, where more detailed instructions are available, including tutorials with short clips.

The XML parser is available in the Settings window. It can be used to parse the XML database file from the HPA website (Fig. 2E). Its unzipped format can be loaded and subsets of the data, based on normal or cancer tissues, can be extracted into an easily readable.cvs format that will contain all the available gene IDs, gene names, antibodies and online image locations. The file will be automatically saved to the same folder as the application.

To demonstrate the use of the application, we set out to identify new and additional immunohistochemical biomarkers for the basal cells of prostate glands. This cell type surrounds the glands in normal tissues but typically disappears in prostate cancer. In histopathology, three markers are routinely used to identify prostate basal cells: CK14, CK5, and P63. Pathologists use these markers to help diagnose prostate cancer as their absence indicates invasiveness [16–19].

Using the XML parser functionality, we downloaded the list of Ensembl IDs for normal prostate tissue. As the scope was to demonstrate the usefulness of the software, we selected a subset of the data from the gene list to analyze. We loaded the list into the AtlasGrabber, selected normal prostate tissue to analyze, and went through the list, saving the genes that showed the staining pattern of basal cells. Again using the AtlasGrabber, we compared with their expression in prostate cancer.

## Results and discussion

We have created an application to enable large-scale, semi-automated analysis of the HPA database. The application was made for the Windows platform. The source code is available on GitHub with a license that allows for free use and further improvements. We have used earlier versions of our software extensively in our research [11, 12] and have identified useful additional refinements, which have been implemented in the current version. The license allows other users to access the source code and implement further functional improvements to their likes.

HPASubC [20] is a previously designed software with a similar aim to facilitate the viewing and analysis of HPA images. However, it only runs on the Linux kernel, requires several python scripts to run, and relies on several dependencies to run, many of them outdated. We found it challenging to run this software, even for technically skilled users. In comparison, our application can easily run on Windows. It also allows for the simultaneous comparison of several windows, allowing the comparison of different tissues and tumor types. It is possible to save genes of interest into separate lists and it does not require the download and storage of a large number of images. It also has an additional feature, the XML parser, that can be used to extract data from the raw database file of the HPA.

Similar information to the HPA can be obtained by employing single-cell analysis (SCA) of tumor tissues. However, with SCA, information of tissue localization and the microenvironment is lost. Therefore, the HPA provides essential additional information that is unique, and as far as we are aware is offered by no other database.

**Fig. 4:**
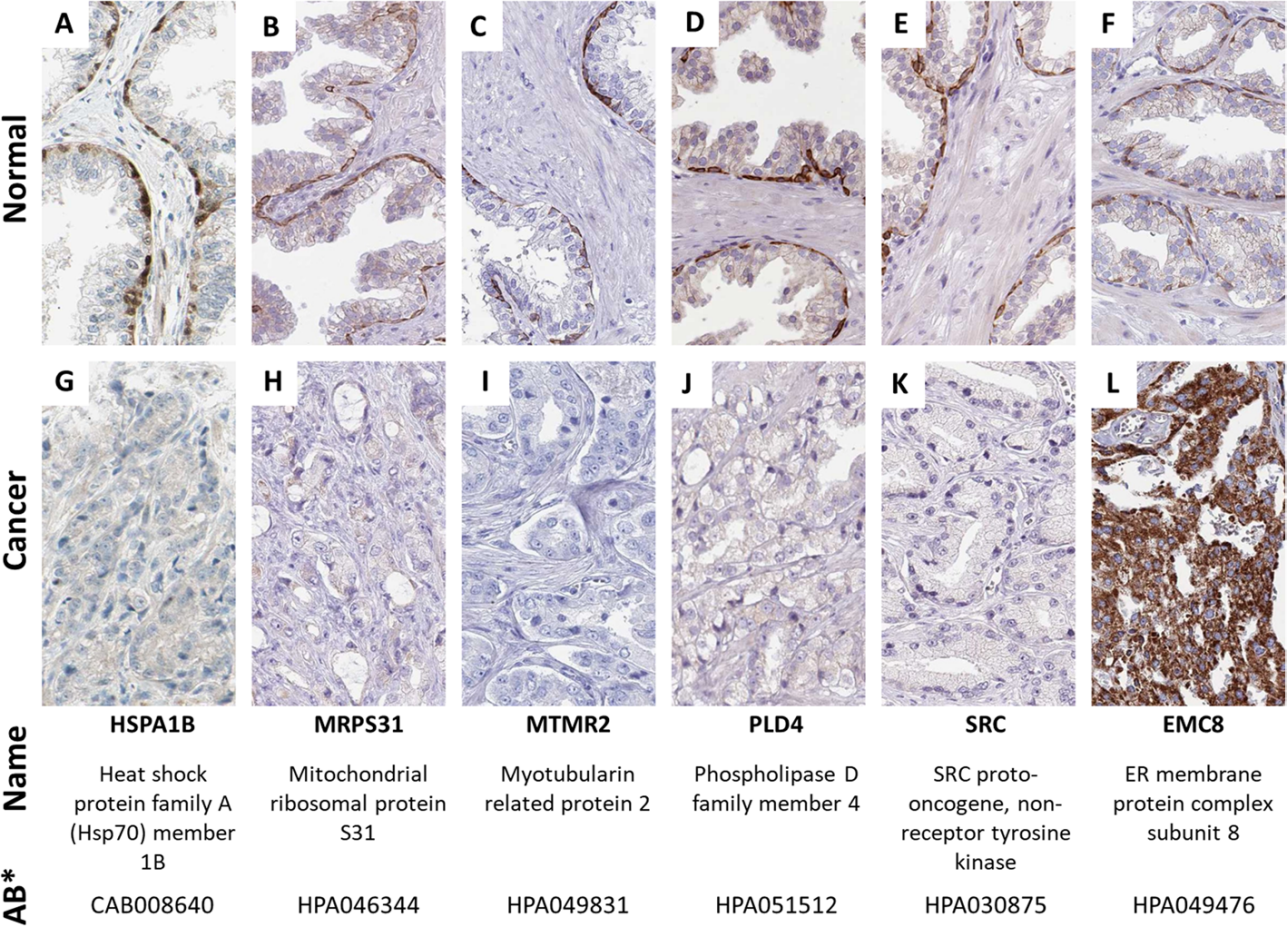
Identification of new biomarkers for the basal cells of the prostate gland. **A**–**F** shows the images of the identified basal cell markers of the prostate in normal prostate tissue, and **G**–**L** their expression in prostate cancer. Each column represents a particular gene and antibody. The table at the bottom shows the gene names and the specific antibody* used. Five of the specific proteins disappear in cancerous tissues (**G**–**K**), while EMC8 (**L**) shows positivity in prostate cancer cells. Image credit: human protein atlas

To demonstrate the usefulness of our software, we performed a brief analysis to find proteins expressed in basal cells of normal prostate tissue. We managed to identify six new immunohistochemical biomarkers expressed in these cell types by analyzing a subset of the images from the HPA, including HSPA1B and SRC (Fig. 4). Analyzing their expression in cancerous tissue shows that they are absent in prostate cancers, with one exception, EMC8, which is also expressed in prostate cancer, although not in basal cells. The other five markers were specifically only expressed in the basal cells.

A limitation to the analysis is the reliability of the antibody specificity, a known challenge in immunohistochemistry. For example, for SRC, two antibodies are targeting the same protein, but only one is specific for basal cells. The other antibody is absent in the normal prostate and expressed in prostate cancer cells. Therefore, we also provide information on the specific antibody that shows the difference we identified for each protein expressed. For each antibody used, the HPA website provides the type of validation used and its overall validation score (supported, validated, or uncertain) [21].

Even though the software greatly increases the ease with which one can analyze a set of genes in the HPA, its limitation is that it still requires the investigator´s input, and the analyses can therefore be time-consuming for larger datasets. Future software should be combined with artificial intelligence approaches to further speed up the analysis using deep learning image classification methods.

Prostate cancer is the most common cancer affecting men worldwide [22]. Stratifying the disease into those who should receive treatment and those who should not is particularly important since it is common to have indolent cancer that might not need to be treated [23–26]. In addition to the TNM score and the PSA levels in the blood, the Gleason score of the cancer histology is used to determine the prognostic risk of the patient. The Gleason Score was established in 1966, and although it has undergone revisions, it remains largely unchanged [27, 28]. In unclear cases, a more detailed analysis is performed using immunohistochemical characterization. Prostate basal cell markers are typically employed, as both the basal cells and the markers are usually downregulated in prostate cancer. Our identified five new markers specific for basal cells in the prostate could potentially be useful in further stratification of prostate cancer to refine and personalize patient treatments.

## Conclusions

We have developed a user-friendly software to facilitate large-scale analysis of the Human Protein Atlas database by organizing images for viewing and scrutinizing based on a predefined gene set. Compared to a software with a similar aim that was published previously, the AtlasGrabber is more user-friendly and with additional functionalities.

Its main utility is that it allows a user to easily analyze a set of genes for their protein expression in different normal and cancerous tissues, and to easily sort interesting candidate genes into separate subsets that can be saved. This will allow users to identify biomarkers specific to a cell, tumor, or tissue type.

To demonstrate its usefulness, we performed an analysis of normal prostate tissues and identified six new biomarkers for basal cells of the prostate. The AtlasGrabber also enables the easy comparison of normal and cancerous tissues. Using this functionality, we compared our identified genes to prostate cancers. This revealed that five of our markers showed no positivity in prostate cancers, while one marker was positive in prostate cancer cells.

The HPA is a rich database making available a wealth of data to scientists. It is freely available, but the difficulty in making large-scale analyses has limited its use so far. Our software will enable researchers to exploit and analyze the images in the database at level with its large capacity. With the ongoing expansion of digital pathology, additional HTP, IHC based tissue repositories will emerge, for which a similar approach can be adopted.

## Availability and requirements

**Project name:** Atlas Grabber.

**Project homepage and source code:** https://github.com/b3nb0z/AtlasGrabber.

**Operating systems:** Windows 8, 10, and 11.

**Programming language:** C#.

**Other requirements:** None.

**License:** GNU GPLv3.

**Any restrictions to use by non-academics:** Users can change or rework the code, but if they distribute these changes/modifications in binary form, they’re also required to release these updates in source code form under the GPL v3 license.

## Data Availability

The datasets analysed during the current study are available in the Human Protein Atlas repository, https://www.proteinatlas.org/download/proteinatlas.xml.gz.

## Abbreviations

HPA: Human protein atlas
ECM: Extracellular matrix
IHC: Immunohistochemistry
HTP: High throughput
SCA: Single cell analysis
